# Histomorphometric Effects of 2% Risedronate Gel on Calvarial Bone Defects in Rabbits 

**DOI:** 10.30476/DENTJODS.2020.82926.1032

**Published:** 2021-03

**Authors:** Shabnam Aghayan, Ahmad Asghari, Pejman Mortazavi, Shirin Marzoughi

**Affiliations:** 1 Dept. of Periodontology, Faculty of Dentistry, Tehran Medical Sciences, Islamic Azad University, Tehran, Iran; 2 Head of Dept. of Clinical Science, Faculty of Specialized Veterinary Sciences, Science and Research Branch, Islamic Azad University, Tehran, Iran; 3 Head of Dept. of Pathology, Faculty of Specialized Veterinary Sciences, Science and Research Branch, Islamic Azad University, Tehran, Iran; 4 Postgraduate Student of Pediatric Dentistry, Dental Research Center, Dept. of Pediatric Dentistry, School of Dentistry, Isfahan University of Medical Sciences, Isfahan, Iran

**Keywords:** Bone Loss, Osteoclast, Rabbit, Risedronate

## Abstract

**Statement of the Problem:**

Alveolar bone resorption associated with periodontal disease is a common finding and generally irreversible. It impairs mastication and causes esthetic problems for patients. Bisphosphonates are the most commonly used antiresorptive agents for bone diseases.

**Purpose:**

Considering the risk of bisphosphonate-related osteonecrosis of the jaw, this study aimed to assess the effect of 2% risedronate gel on calvarial bone defects in rabbits.

**Materials and Method:**

In this animal study, critical-size defects of 8mm were created in the calvaria of 20 New Zealand white rabbits. In group 1 (n=10), 2% risedronate gel was applied into the right side defect while the left side defect remained empty and served as control. In group 2 (n=10), placebo gel was applied into the right side defect, while the left side defect remained empty and served as control. Five rabbits in each group were sacrificed at 1month and the remaining five at 2 month, post-operatively, and tissue samples were collected for histomorphometric analysis. Histomorphometric assessments included bone fill, degree of inflammation, number of osteoblasts, number of osteoclasts, and foreign body reaction at the site. Data were statistically analysed using SPSS version 25 via the Dunn test and Kruskal-Wallis test.

**Results:**

No bone remodeling was noted in any group at 1 month. The risedronate group showed significantly higher bone fill than the other groups after 2 months (*p*= 0.016). At 2 months, the number of osteoblasts was significantly higher in the risedronate group (*p*< 0.05). The groups were not significantly different in terms of inflammation score at 1 (*p*= 0.31) or 2 (*p*=0.69) months. Foreign body reaction was not observed in any group at any time point. No osteoclast was detected in any group at any time point.

**Conclusion:**

Risedronate gel showed superior efficacy with regard to regeneration of rabbit calvarial bone defects compared to the placebo and control groups.

## Introduction

Alveolar bone resorption associated with periodontal disease is a common finding and generally irreversible. It impairs mastication and causes esthetic problems for patients. The efficacy of bisphosphonates to prevent or slow down the process of bone resorption has been previously documented [ 1 ]. Bisphosphonates are the most commonly used antiresorptive agents for treatment of bone diseases. They enhance bone remodeling by directly targeting the osteoclasts [ 2 ]. Bisphosphonates were first synthesized in 1897 but the first report regarding their biological effects was published in 1969 [ 3 ]. Zoledronate is the most potent bisphosphonate, which was approved by the Food and Drug Administration (FDA) in 2011 [ 4 ]. Despite its constructive role in bone remodeling, zoledronate may also have side effects such as osteonecrosis of the jaw, the occurrence of which depends on the method of usage and duration of treatment [ 5 ]. The prevalence of osteonecrosis of the jaw varies from 0.01% to 0.04% in oral intake and 0.8% to 1.2% in intravenous administration of zoledronate [ 6 ]. In 2003, Marx *et al*. [ 7 ] reported the first case of osteon-ecrosis of the jaw as a result of treatment with zoledronate.

Risedronate has an antiresorptive effect 5000 times greater than that of etidronate and 5 times greater than that of alendronate with no cytotoxicity [ 1 , 8 ]. Cetinkaya *et al*. [ 13 ] evaluated the effect of risedronate on bone resorption and angiogenesis and concluded that short-term treatment with risedronate can effectively prevent bone resorption due to periodontitis. Khajuria *et al*. [ 16 ] reported the positive effect of chitosan-based risedronate/ zinc-hydroxyapatite intra-pocket dental film on proliferation and differentiation of osteoblasts. Considering its higher efficacy and shorter treatment period compared to other bisphosphonates, risedronate may show promising results in regeneration of bone defects [ 1 ]. Thus, this study aimed to assess the efficacy of 2% risedronate gel for regeneration of rabbit calvarial bone defects.

## Materials and Method

This animal study evaluated 20 male New Zealand rabbits between 6 to 7 months old, weighing 2 to 2.5 kg. They were obtained from the Department of Animal Sciences, Pasteur Institute, Iran, and were kept in separate cages. To ensure their optimal health status, all rabbits underwent a comprehensive clinical examination and received anti-parasitic medication as a precautionary measure. For the purpose of acclimation, no tests were conducted on rabbits during the first week and their environmental conditions remained unchanged. They were kept at 22±2° temperature and 70% relative humidity, with 12-hour dark/12-hour light cycle to help keep their normal circadian rhythm. They were fed in regular laboratory plates and had ad libitum access to drinking water. This study was conducted in accordance with the ethics and protocols of the American Veterinary Medical Association. 

### Preparation of 2% risedronate gel

The risedronate powder is white, odorless, and water-soluble with a melting point of 252°C to 262°C. We used risedronate powder, 934 Carbomer powder, tri-ethanol amine, absolute alcohol, and sterile distilled water to produce risedronate gel. For the production of 2% risedronate gel, 2 g of risedronate powder was dissolved in 60 cc of sterile distilled water heated to 50°C; 20 cc of ethanol was also added and mixed. Next, 3 g of Carbomer powder (for gelation) was added to the glass container and stirred. Since maximum stability of this product is at a pH of 7.5, the pH was adjusted by using a small amount of triethanolamine. The final volume was then reached to 100 g using distilled water and the mixture was allowed to gel and hydrate; 2w/w% risedronate gel was obtained as such. Next, the product was UV-sterilized for 60 minutes. Alcohol was used in this process to prevent microbial contamination.

### Surgical procedure

The rabbits were randomly divided into two groups of cases and placebo (n=10). The animals were refrained from eating for 4 hours and drinking for 2 hours prior to the surgical procedure. In order to anesthetize the rabbits, 10% ketamine and 2% Xylazine were injected intramuscularly in 40 mg/kg and 10 mg/kg of body weight doses, respectively. After reaching adequate depth of anesthesia, the surgical site (skull) was shaved, scrubbed and locally anesthetized using 2% lidocaine. The surgical site was then draped. Afterwards, a 10 cm incision was made at the skull midline from the center point of the ears towards the forehead and a full-thickness anterior-posterior flap was elevated. Once the skull was exposed, the periosteum was elevated by a periosteal elevator. Next, two critical size defect (holes) with 8 mm diameter were created in the calvaria of each rabbit [ 17 ], one in the right and the other in the left side using a drill (XST083025 Dask, Dentium Advanced Sinus Kit, Dentium, Seoul, Korea). The holes were drilled under copious saline irrigation to prevent overheating. The area was then washed with saline and cleaned from bone chips. In the case group rabbits, the right hole was filled with 2% risedronate gel while the left hole remained empty as the negative control. In the placebo group rabbits, the right hole was filled with the placebogel while the left hole remained empty as the negative control. 

After filling the critical-size defect, the periosteum and the calvarial skin were sutured using 4-0 vicryl and 3-0 nylon sutures, respectively. After regaining consciousness, the rabbits were transferred to their cages and were provided with food and water. All rabbits received an intramuscular injection of 20 mg/kg cefazolin prophylactically and also received analgesics for 5 days, postoperatively. The rabbits were monitored on a daily basis for any swelling or signs of inflammation around the defect site, as well as the status of sutures, signs of infection and discharge. The sutures were removed after 10 days.

The duration of the study was 2 months [ 17 ]. At the end of each month, five rabbits from each group were sacrificed painlessly by injection of sodium thiopental. Then, the surgical site was incised and the calvaria was resected and sent to a laboratory for histomorphometric analysis by one calibrated pathologist. 

### Sample preparation for histomorphometric assessment

The soft tissue around the calvaria was fully dissected using a #22 surgical scalpel. The entire forehead as well as the superior rim of the orbit was dissected and soft tissues were completely removed. The orbital rim helped to determine the anterior-posterior direction of the defects. Samples were separately kept in 10% formalin for 2 weeks until the fixation was complete. Then, they were placed in 10% formic acid solution for 4 weeks. During this period, the softening of samples was closely monitored daily. This was done to decalcify the samples for easier sectioning by a microtome. The samples were temporarily transferred from formic acid to formalin every 2 days in order to fix the decalcified regions. Neutralization was performed by immersing the samples in 20% lithium carbonate solution for 5 minutes to enhance the quality of staining.

Each sample was then coded using codes consisting of a letter and a number. The letter represented the group that the rabbit belonged to (case or placebo) and the number represented the time of sacrifice (1 or 2 months). Defects in the case group were coded A and B and those in the placebo group were coded C and D. For example, A1 was the first rabbit in the case group, which was sacrificed at 1 month, and B1 was the first rabbit in the case group, which was sacrificed at 2 months. Then, each defect was cut into halves at the centerline in anterior-posterior direction. The cutting edge, which represented the midline, was marked with Indian ink, and after dehydration with 70% to 100% ethanol, each specimen was embedded in paraffin blocks from the marked side. Two slices were cut out of each paraffin block with 5 µ thickness and stained with hematoxylin and eosin. The samples were then inspected under an optical microscope (Euromex, Holland) at ×64 and ×160 magnifications. The histomorphometric analysis included assessment of the amount of bone fill (newly formed viable bone), bone remodeling, and number of osteoblasts, number of osteoclasts and presence/absence of foreign body reaction. These parameters were separately evaluated and recorded in a checklist for each defect by a calibrated pathologist. The qualitative variables, evaluated by the pathologist, included presence/absence of inflammation, foreign body reaction, and the amount of newly formed bone. The quantitative variables included the number of osteoblasts and the number of osteoclasts, which were measured by taking photographs of each defect during the course of study. For this purpose, a digital camera (DP12; Olympus, Japan) with ×20 magnification was used. Then, the Magic Wand tool of the Adobe Photoshop software was used to study regions around the defects to determine the areas of new bone formation and biomaterial, which had similar color characteristics to those of bone and could not be finely distinguished with the naked eye. Using the Histogram command, the number of pixels in each region was measured and recorded [ 17 ]. Presence/absence of inflammation was scored as Score 0: absence of inflammatory cells, Score 1: presence of inflammatory cells <25%, Score 2: presence of inflammatory cells between 25% to 50%, Score 3: presence of inflammatory cells between 50% to 75%, and Score 4: presence of inflammatory cells >75%. 

Bone remodeling was scored as Score 0: no remodeling, Score 1: remodeling <25%, Score 2: remodeling between 25% and 50%, Score 3: remodeling between 50% and 75%, and Score 4: remodeling >75%.

The number of osteoblasts was scored as Score 0: absence of osteoblasts, Score 1: <10 osteoblasts in each field, and Score 2: presence of 10 to 20 osteoblasts in each field and Score 3: >20 osteoblasts in each field 

The bone fill was scored as Score 0: empty defect (no bone fill),Score 1: presence of fibrotic tissue only, Score 2: presence of high amounts of fibrous tissue and small amounts of cartilage, Score 3: presence of equal amounts of cartilage and fibrous tissue, Score 4: presence of high amounts of cartilage and small amounts of fibrous tissue, Score 5: presence of cartilage only, Score 6: presence of high amounts of cartilage and small amounts of immature bone, Score 7: presence of equal amounts of cartilage and immature bone, Score 8: presence of significant amounts of immature bone and small amounts of cartilage, Score 9: healing with immature bone, and Score 10: healing with mature bone.

The collected data were analyzed using SPSS version 25 (SPSS Inc., IL, USA) via the Dunn test and the Kruskal-Wallis test. 

## Results

**Figure 1:**
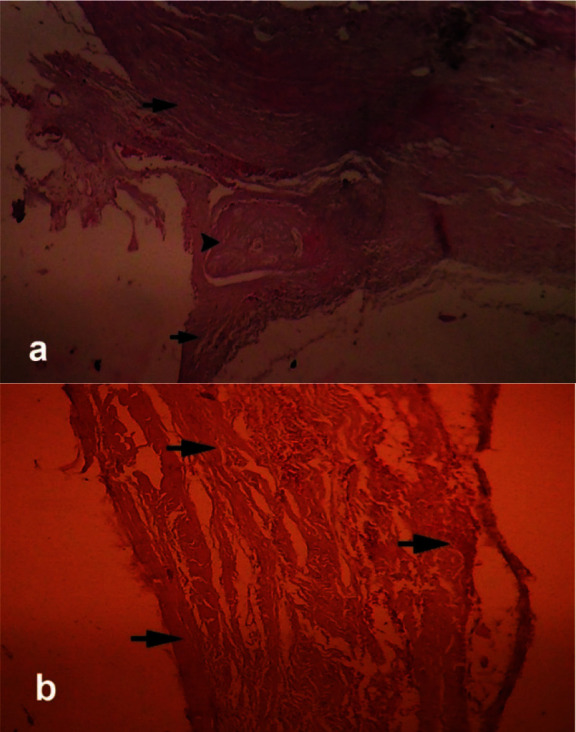
defect filled with connective tissue only (arrow); no cartilaginous callus is present, (H&E, ×160 magnification)

Table 1 presents the inflammation scores in the two groups at 1 and 2 months. The groups were not significantly different in terms of inflammation score at 1 (*p*= 0.31) or 2 (*p*= 0.69) months (Table 1). No bone remodeling was noted in any group at 1 month (Table 2) shows the remodeling score of the groups at 2 months. No significant difference was noted between the groups in bone remodeling at any time point (*p*= 1.00 at 1 month and *p*= 0.09 at 2 months). Osteoblasts were not observed in any group at 1 month (Table 3). Table 2 presents the number of osteoblasts in the groups at 2 months. No significant difference was noted in the number of osteoblasts at 1 month between the groups (*p*= 1.00). No significant difference was noted between the placebo and control groups in the number of osteoblasts at 2 months (*p*= 0.151) but the difference between the case and control groups was significant in this respect (*p*= 0.008). The difference between the case and placebo groups was also significant (*p*= 0.016) such that the number of osteoblasts in the case (risedronate gel) group was significantly higher than that in the control and placebo groups. Table 4 shows the bone fill score of the groups at 1 and 2 months. No significant difference was noted in bone fill among the groups at 1 months (*p*= 0.22). There was no significant difference in this regard between the placebo and control groups at 2 months (*p*= 0.151). However, the difference in this respect was significant between the case and control groups (*p*= 0.008). The difference between the case and placebo groups was also significant (*p*= 0.016) such that the bone fill was significantly greater in the case group compared to the placebo and control groups. No osteoclast was detected in any group at any time point. Foreign body reaction was not observed in any group at any time point either. Figure 1a and b shows microscopic images of tissue specimens in the groups.

**Table 1:** Inflammation score of the groups at 1 and 2 months

Time				Inflammation		Total
				1.00	2.00	
1.00	Control	Group	Placebo	4	1	5
			Control	4	1	5
		Total		8	2	10
	Case	Group	Drug	2	3	5
			Control	4	1	5
		Total		6	4	10
2.00	Control	Group	Placebo	4	1	5
			Control	4	1	5
		Total		8	2	10
	Case	Group	Drug	3	2	5
			Control	4	1	5
		Total		7	3	10

**Table 2:** Bone remodelling score of the groups at 2 months

Time				Remodeling					Total
				.00	1.00	2.00	3.00	4.00	
2.00	Control	Group	Placebo		4	1	0		5
			Control		1	4	0		5
		Total			5	5	0		10
	Case	Group	Drug		0	0	4	1	5
			Control		2	3	0	5
		Total			0	2	7	1	10

**Table 3:** Number of osteoblasts in the groups at 2 months

Time				Number of osteoblasts			Total
				0	1	2	
2.00	Control	Group	Placebo	0	4	1	5
			Control	0	1	4	5
		Total		0	5	5	10
	Case	Group	Drug	0	1	4	5
			Control	2	3	0	5
		Total		2	4	4	10

**Table 4:** Bone fill of the groups at 1 and 2 months

Time				Fill								Total
				1.00	2.00	3.00	5.00	6.00	7.00	8.00	9.00	
1.00	Control	Group	Placebo	4	1							5
			Control	2	3							5
		Total		6	4							10
	Case	Group	Drug	0	4	1						5
			Control	2	3	0						5
		Total		2	7	1						10
2.00	Control	Group	Placebo				0	4	1			5
			Control				0	1	4			5
		Total					0	5	5			10
	Case	Group	Drug				0	0	1	3	1	5
			Control				2	3	0	0	0	5
		Total					2	3	1	3	1	10

## Discussion

This study histomorphometrically evaluated the efficacy of 2% risedronate gel for bone regeneration in rabbit calvarial bone defects during 2 months.

The results showed that the case (risedronate) group showed significantly higher bone fill than the control group (*p*< 0.05). In addition, the number of osteoblasts was significantly higher in the risedronate gel group at 2 months compared to other groups (*p*< 0.05). 

Foreign body reaction was not observed in any group during the study period. Bisphosphonates containing nitrogen are specifically known for their bone loss inhibitory effect. Risedronate is among the most renowned nitrogen-containing bisphosphonates, which exerts its inhibitory effect by binding to hydroxyapatite crystals in bone and inhibiting the activity of adjacent osteoclasts [ 9 ]. Bisphosphonates inhibit the synthesis of farnesyl diphosphate in mevalonic acid pathway and consequently induce apoptosis of osteoclasts [ 9 ]. Risedronate causes apoptosis of macrophages in relatively lower concentrations than other bisphosphonates such as alendronate and pamidronate in vitro [ 10 ]. Risedronate prevents osteoblast-stimulated osteoclast differentiation, and this reduction of differentiation of osteoclasts is not due to toxicity [ 9 ]. Risedronate also suppresses the RANKL-mediated osteoclast differentiation, inhibits RANKL-dependent c-Fos and NFATc1 expression and suppresses RANKL-induced p38 phosphorylation [ 9 ]. In general, risedronate inhibits osteoclast differentiation directly and indirectly. Bisphosphonates present in bone are released into the Howship’s lacunae and directly enter into osteoclasts by endocytosis. Next, the osteoclasts lose their ruffled border and their cytoskeleton is gradually degraded. Thus, their destructive activity is impaired and apoptosis is induced [ 11 ]. This process occurs as the result of inhibition of farnesyl diphosphate synthase [ 12 ]. Risedronate is used for treatment of post-menopausal and elderly osteoporosis, rheumatoid arthritis, Paget’s disease, osteomyelitis, and chronic periodontitis [ 9 ].

Cetinkaya *et al*. [ 13 ] evaluated the effect of risedronate on bone resorption and angiogenesis and concluded that short-term treatment with risedronate can effectively prevent bone resorption due to periodontitis.However, long-term treatment with risedronate may damage the bone and impair angiogenesis. They systemically administered risedronate for 3 weeks and observed favourable results; whereas, we locally applied risedronate gel into bone defects as single dose and observed favourable results. The reason for our favourable results may be the application of proper concentration of risedronate in gel form in contact with bone. Compared to systemic administration, local application has advantages such as fewer side effects and superior biocompatibility. The main advantage of local application of medications compared to their systemic administration is the use of much lower dosage at the defect site to produce the desired therapeutic outcome [ 14 ]. In this study, the results showed a significant difference in bone fill between the case and control groups and between the case and placebo groups such that the bone fill in the case group was significantly higher than that in the control and placebo groups. Sharma *et al*. [ 15 ] evaluated the effect of clinical application of 1% alendronate gel for treatment of chronic periodontitis. They concluded that 1% alendronate gel decreased the pocket depth, increased clinical attachment level, and enhanced the bone fill compared to the placebo gel. In our study, the case and control groups were significantly different in number of osteoblasts at 2 months. The difference between the case and placebo groups was also significant such that the number of osteoblasts in the case group was higher than that in the control and placebo groups. Khajuria *et al*. [ 16 ] reported the positive effect of chitosan-based risedronate/zinc-hydroxyapatite intra-pocket dental film on proliferation and differentiation of osteoblasts. They concluded that preservation of alveolar bone by this compound might be due to its inhibitory effect on bone loss by inhibiting the activity of osteoclasts [ 16 ]. They also reported the positive effect of chitosan on proliferation and maturation of osteoblasts [ 16 ]. We also used risedronate locally; however, we produced a pure gel with no base or polymer; thus, our results can be totally attributed to pure risedronate. However, many questions remain unanswered regarding the mechanism of action of risedronate gel with regard to the proliferation of osteoblasts and osteogenesis, which call for further investigations on this topic.

Risedronate gel showed superior efficacy with regard to regeneration of rabbit calvarial bone defects compared to the placebo and control groups. However, further animal studies with larger sample size and longer follow-ups are required to cast a final judgment in this respect.

## Conclusion

Risedronate gel showed superior efficacy with regard to regeneration of rabbit calvarial bone defects compared to the placebo and control groups. 
